# Aquaporins, Colonic Water Transport, and Functional Constipation

**DOI:** 10.3390/ijms272110019

**Published:** 2026-11-09

**Authors:** Kai Zheng, Rui Li, Wenyue Ma, Daqing Sun

**Affiliations:** Department of Pediatric Surgery, Tianjin Medical University General Hospital, Tianjin 300052, China; kzhengkz@126.com (K.Z.); lirui@tmu.edu.cn (R.L.); mwy19980322@163.com (W.M.)

**Keywords:** aquaporins, colonic water transport, functional constipation, slow-transit constipation, AQP3, gut microbiota

## Abstract

Functional constipation (FC) is a common disorder of gut–brain interaction whose clinical signature, hard, infrequent stools, points to disturbed colonic water handling. Aquaporins (AQPs) provide the transcellular route for transepithelial water movement in the colon, and evidence links AQP dysregulation to FC and slow-transit constipation (STC). This narrative review synthesizes human tissue studies, animal models, and in vitro experiments on colonic AQPs in constipation. Limited human tissue studies report segment-specific changes in AQP abundance. AQP3 is upregulated in the ascending colon, whereas AQP9 is downregulated in the descending colon. AQP4 is elevated in STC and correlates with stool consistency and colonic transit time. AQP8 is upregulated and undergoes osmotic-stress-driven translocation to the apical (luminal) membrane. A broader mucosal survey additionally found reduced AQP1, AQP7, AQP10, and AQP11 in FC. Experimental models add mechanistic insight but reveal no conserved direction of AQP change. Because AQPs conduct water passively down osmotic gradients, channel abundance cannot be equated with channel-mediated water flux. Regulatory pathways, including macrophage-derived prostaglandin E2 (PGE2), cytokines, microbial metabolites, and enteric neurotransmitters, converge on the AQP network, with AQP3 as a recurrent node. The available evidence supports an association between AQP dysregulation and the constipated phenotype without establishing causality. Functional readouts of transepithelial water movement in patients are needed before AQP modulation can be translated into therapy.

## 1. Introduction

Functional constipation (FC) is a disorder of gut–brain interaction defined by the Rome IV criteria as persistently difficult, infrequent, or incomplete defecation that cannot be attributed to an underlying structural, biochemical, or psychiatric disorder [1].

FC is among the most prevalent gastrointestinal disorders. A meta-analysis of community-based studies estimates that chronic (idiopathic) constipation affects approximately 14% of adults, with higher rates in women and among older adults [2]. In the broader landscape of disorders of gut–brain interaction, the Rome Foundation Global Study found that more than 40% of adults met criteria for at least one functional gastrointestinal disorder [3]. That study enrolled more than 73,000 adults across 33 countries. Chronic constipation is a phenotype that overlaps with but is broader than FC, as it also encompasses secondary and medication-related causes. Physiologically, chronic constipation is heterogeneous, and patients with FC are no exception. It can be further categorized into normal-transit constipation, defecatory disorders, and slow-transit constipation (STC). Among these, STC, defined by delayed colonic transit in the absence of anorectal dysfunction, is the least common but the most treatment-refractory subtype and accounts for a disproportionate share of surgical referrals [1,4].

The clinical signature of FC indicates a disturbance in colonic water-handling mechanisms. Approximately 1.5–2 L of fluid enters the colon each day, and the healthy colon absorbs most of it. Normal stool water content (roughly 70–75%) therefore reflects a delicate balance between epithelial absorption and secretion [5,6]. In FC, excessive absorption, deficient secretion, or both would desiccate the stool, yet the molecular mechanisms that maintain this balance, the machinery that conventional therapies engage, remain incompletely understood. These therapies act through distinct mechanisms: osmotic laxatives modulate luminal osmolarity, stimulant laxatives and prokinetics enhance motility and secretion, and secretagogues promote epithelial fluid secretion. Mechanistic studies in rodents show that the stimulant laxatives bisacodyl and sennoside A reduce colonic AQP3 expression [7,8]. Morphine, the archetypal trigger of opioid-induced constipation, elevates colonic AQP3 expression in rodent models, a finding consistent with, but not alone sufficient to demonstrate, enhanced colonic water absorption [9]. What is missing is not evidence that AQPs matter, but a functional framework for interpreting AQP changes in patients and for designing therapies around these channels [10,11].

Aquaporins (AQPs) are small integral membrane proteins that function as water-selective channels [12]. The thirteen mammalian AQPs (AQP0–AQP12) can be classified into three groups. The orthodox channels (AQP0, AQP1, AQP2, AQP4, AQP5, AQP6, and AQP8) are primarily water-permeable, although AQP6 is unusual in that it also conducts anions in a pH-sensitive manner. The aquaglyceroporins (AQP3, AQP7, AQP9, and AQP10) additionally transport glycerol and other small solutes. The unorthodox channels (AQP11 and AQP12) are predominantly localized in intracellular membranes and have the least well-defined transport roles [13,14]. AQPs assemble as homotetramers, with each monomer forming an hourglass-shaped pore. These pores permit rapid, passive water movement down osmotic gradients and, in some compartments, hydrostatic gradients. The channels accelerate transepithelial water flux but do not themselves generate the driving force [12,15]. The colon expresses a distinctive repertoire, including AQP1, AQP3, AQP4, AQP7, AQP8, AQP9, AQP10, and AQP11, whose polarized distribution across the epithelium defines the transcellular route of water absorption [16].

Direct human evidence now links this repertoire to FC. In patients with FC, mucosal AQP3 is increased in the ascending colon and AQP9 is reduced in the descending colon [17]. AQP4 is elevated in STC and correlates with stool consistency and colonic transit time [18]. Under osmotic stress, AQP8 is redistributed from the cytoplasm to the apical (luminal) membrane, a response involving p38/JNK mitogen-activated protein kinase (MAPK) signaling in cultured colonocytes [19]. In parallel, a rapidly growing experimental literature, largely involving rodent models of chemically induced constipation and probiotic, botanical, and dietary interventions, reports coordinated changes in colonic AQP expression [20,21,22]. The direction of these changes, however, is far from consistent. That inconsistency is itself informative. The functional outcome of a change in channel abundance may depend on the osmotic and electrochemical context in which the channel operates.

Earlier studies have mapped AQP expression along the gastrointestinal tract and have identified AQP3 as a candidate colonic water channel, thereby highlighting it as a potential therapeutic target in both diarrheal and constipated conditions [10,15,16]. Building on this foundational work, this review provides a critical synthesis of the evidence linking AQPs to FC. It integrates human data on colonic AQP expression, insights from experimental constipation models, and the regulatory pathways that converge on the AQP network, including inflammation, microbial metabolites, and enteric neurotransmission. The review then examines the therapeutic implications of these findings.

## 2. Methodology

This review is a narrative synthesis. We searched PubMed/MEDLINE, Scopus, Web of Science, and Google Scholar from database inception through September 2026. The search combined terms for aquaporins (e.g., ‘aquaporin’, ‘AQP1’–‘AQP11’), colonic water handling and constipation (e.g., ‘colonic water transport’, ‘transepithelial water flux’, ‘functional constipation’, ‘slow-transit constipation’), together with terms for upstream regulation (inflammation, prostaglandin E2 (PGE2), short-chain fatty acids, gut microbiota, enteric neurotransmission) and interventions (laxatives, secretagogues, probiotics, natural products, electroacupuncture). Titles and abstracts were screened for relevance, and full texts of eligible articles were then evaluated. Reference lists of selected studies were manually reviewed to identify additional relevant publications.

We included original research articles, covering human tissue studies, animal models, and in vitro experiments, that reported AQP expression, localization, or function in the colon in the context of FC, STC, or related experimental models, as well as studies examining regulatory pathways and interventions relevant to AQP modulation. Review articles were used to provide contextual background and support the interpretation of findings. Studies were excluded if they addressed AQPs outside the gastrointestinal tract and lacked relevance to the colon, did not report outcomes relevant to constipation, or were not full-text publications (e.g., conference abstracts). Data were extracted from the included studies on species and experimental model, tissue and cell type, colonic segment, AQP isoform, direction and magnitude of expression change, and presence of a functional readout for water transport. The findings were synthesized thematically according to three questions: what the human evidence establishes, what experimental models add, and how regulation and therapy relate to the colonic AQPs. Because the aim was thematic integration rather than quantitative pooling, no formal risk-of-bias scoring or meta-analysis was performed.

## 3. AQPs and Colonic Water Transport

### 3.1. The Colonic AQP Repertoire

The human and rodent colons express a defined set of AQP isoforms whose cellular localization shapes the route of transepithelial water movement. AQP3 is abundant in colonic surface epithelial cells, but its membrane-domain distribution remains unsettled and varies by species and detection method. Reports on the human colon have variously described apical and basolateral predominance, whereas most rodent studies have localized AQP3 to the basolateral membrane [23,24,25,26]. The functional significance of this species difference remains unresolved and argues against assuming a single membrane destination when modeling transcellular water flow. AQP8 is concentrated in subapical cytoplasmic vesicles of colonic absorptive cells, a distribution compatible with regulated insertion into the apical membrane [19,23,27]. AQP1 is expressed predominantly in colonic endothelial cells and, in some species, in glial cells of peripheral nerve plexuses, including enteric plexuses of the esophagus [16,28]. AQP4 is found in the colonic mucosa and in enteric neurons of the myenteric and submucosal plexuses [29]. Its distribution in the human colon is less clearly defined [30]. AQP7 and AQP9 are expressed at lower levels [16,19], with AQP9 associated with a mucus-secreting goblet-cell subset in the small intestine and with goblet cells in the colonic mucosa [17,31]. AQP10 has been hypothesized, on the basis of sequence and structural analyses rather than experimental demonstration, to localize within the apical (glycocalyx) compartment of the human intestinal epithelium [26]. Its expression in the human colon and its functional relevance to colonic water transport remain uncertain. AQP11 is present at the transcript level in the colonic mucosa but is reported predominantly in intracellular membranes [16].

**Figure 1:**
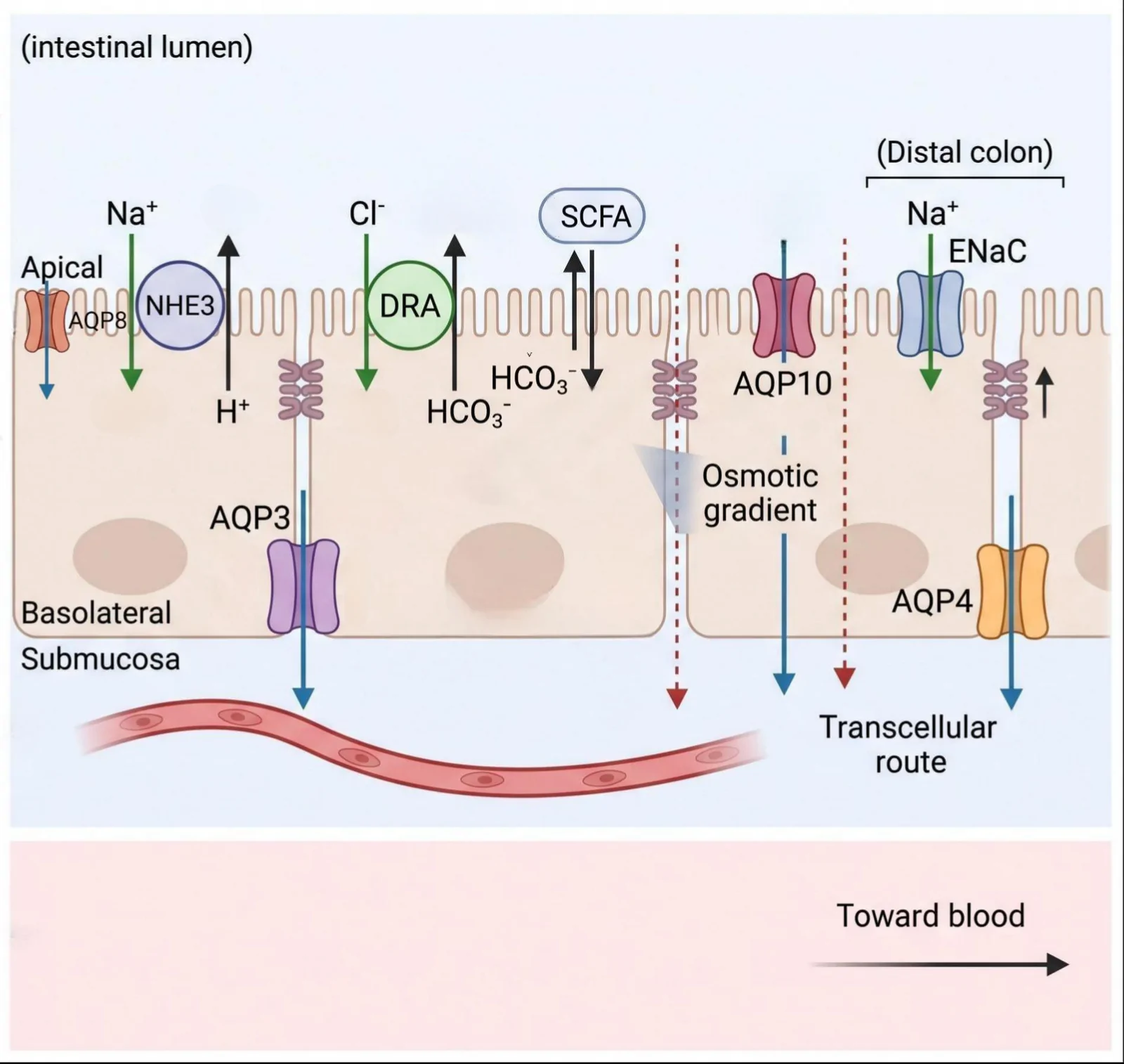
Solute-coupled water absorption across the colonic epithelium. Apical Na^+^/H^+^ exchange (NHE3), Cl^−^/HCO_3_^−^ exchange (DRA), and, in the distal colon, electrogenic Na^+^ absorption via the epithelial sodium channel (ENaC) collectively create the osmotic gradients that drive water movement across the epithelium. Short-chain fatty acids (SCFAs) are absorbed in exchange for bicarbonate. AQPs provide the transcellular route through which water follows these gradients. Abbreviations are listed at the end of the manuscript.

Because net water absorption depends on channels acting in concert across both membrane domains, measuring any single isoform captures only a fraction of the transport system. Caution is also required when extrapolating rodent findings to the human colon, because the membrane destination of key isoforms, notably AQP3 and AQP4, shifts between species. Table 1 summarizes the colonic AQP repertoire, and Figure 1 depicts the main features of this organization schematically.

### 3.2. Solute-Coupled Water Absorption

Colonic water absorption follows solute transport: solute gradients supply the driving force, and water moves osmotically behind them. The dominant processes are electroneutral NaCl absorption, consisting of apical NHE3 coupled to Cl^−^/HCO_3_^−^ exchange, and, in the distal colon, electrogenic Na^+^ absorption through ENaC [5,6]. SCFAs, produced by microbial fermentation, are absorbed in protonated form or in exchange for bicarbonate, and their uptake is tightly coupled to Na^+^ and water movement [32]. Water follows these solutes osmotically, and AQPs provide the transcellular route that accelerates osmotic equilibration across the epithelium. AQP-mediated flux is therefore set by the prevailing solute gradients. A channel cannot create a gradient. It can only conduct water down one that already exists.

This framework reconciles observations that would otherwise appear paradoxical. An increase in AQP3 abundance should enhance absorption only when the transepithelial osmotic driving force is directed from lumen to blood. Under secretory conditions, the same channels would conduct water in the opposite direction. Interventions that alter ion transport can change net water movement without any change in channel abundance. Conversely, AQP modulation without a parallel change in solute transport may have little effect on net flux [32,33]. In the constipated colon, prolonged transit extends the time available for solute-coupled absorption, allowing this machinery to operate at high efficiency. The AQP network then determines how completely the lumen is dehydrated.

The colonic epithelium is moderately tight compared with the small intestine, although it is far leakier than renal collecting-duct epithelia. The quantitative split between transcellular and paracellular water movement across the colonic mucosa remains incompletely defined. A substantial fraction of water transport is believed to proceed paracellularly, driven by the same solute gradients and by solvent drag [5,15]. The transcellular component, in turn, is not carried by AQPs alone, because the lipid bilayer itself has a finite water permeability. What the available evidence supports is therefore a more modest claim: AQPs accelerate the transcellular limb of colonic water transport. The physiological impact of a change in AQP abundance should be greatest when the transcellular limb carries a large share of net flux, for example when solute-coupled absorption is brisk and the epithelium is intact. Transcript levels and immunoblot densitometry measure the total protein pool and cannot distinguish the membrane fraction from intracellular pools, so a change in AQP abundance acquires functional meaning only in the context of localization data, which are reported in only a few of these studies. In their absence, and without direct measurement of water flow, an abundance change does not by itself establish the direction or magnitude of net water transport.

## 4. AQPs in Patients with FC

### 4.1. Expression Studies in the Colonic Mucosa

Direct human data linking AQPs to FC remain sparse and are, for the most part, limited to abundance and localization rather than to water flux. In an early systematic study, immunohistochemistry and immunoblotting of colonic mucosa from 45 patients with FC and 21 controls showed that AQP3 was upregulated in the ascending colon and AQP9 was downregulated in the descending colon [17]. This segmentally discordant pattern argues against a uniform, whole-colon response. In a separate cohort of 45 patients with STC, high AQP4 expression in the ascending colonic mucosa (36 of 45 patients) was associated with dry, hard stool (Bristol types I–II) and a longer colonic transit time (127.3 ± 28.2 h versus 64.2 ± 12.9 h in the low-expression group) [18]. However, because these studies measure channel protein rather than water flux, they cannot establish whether the AQP change was causal or correlative. An additional human study examined the AQP panel more broadly. In colonic biopsies from patients with FC, AQP3 and AQP8 were upregulated, whereas AQP1, AQP7, AQP9, AQP10, and AQP11 were downregulated relative to controls. Additionally, AQP8 was redistributed from the cytoplasm to the apical (luminal) membrane [19]. The constipated mucosa does not exhibit a single isolated AQP change but rather a broader shift in the water-channel repertoire. This finding cautions against interpreting any one isoform in isolation.

### 4.2. Transcriptomic and Single-Cell Analyses

Beyond candidate-gene studies, unbiased approaches have begun to position AQPs within the molecular landscape of STC. RNA-sequencing of colonic tissue identified AQP8 among the differentially expressed genes common to STC and colorectal cancer datasets and proposed it as a potential molecular link in the transition from STC to colorectal cancer [34]. The hypothesis that prolonged colonic transit and malignancy may share an epithelial water channel is thought-provoking, yet it arises from the intersection of public datasets rather than from prospective longitudinal measurements. Single-cell and long non-coding RNA profiling have further delineated a pro-inflammatory stromal–immune microenvironment in STC, marked by *XCL2*^+^ CD8^+^ T-cell–stromal crosstalk [35] and immune-related gene networks [36]. This microenvironment provides a plausible upstream context in which AQP dysregulation occurs.

### 4.3. What the Human Data Can and Cannot Establish

The human data support an association between AQP dysregulation and the constipated phenotype but leave its causal direction unresolved. The scope of this limitation bears directly on clinical relevance. The FC and STC cohorts in which segment-specific AQP changes were first described each comprised 45 patients and were each single-center. These studies were reported between 2008 and 2010 [17,18]. Additionally, the 2023 study describing AQP8 translocation in FC did not report cohort sizes in its abstract [19]. These numbers support hypothesis generation, not prevalence claims. They cannot be pooled because the studies differ in colonic segment, assay, and diagnostic label. The animal data pose a separate extrapolation problem. Loperamide- and diphenoxylate-induced models reproduce delayed transit and low fecal water content, yet they represent drug-induced states rather than the clinical syndrome. Moreover, the direction of the AQP response varies between such models, and they are therefore best interpreted as proof-of-concept rather than as evidence of clinical phenomena. Future studies likely to advance the field would measure electrolyte and fluid transport in patient tissue (e.g., ion flux in Ussing chambers) and would pair these measurements with the membrane localization of AQP3 and AQP8. None of the reviewed human studies has provided this combination.

## 5. Experimental Models of FC

### 5.1. Model-Dependent Changes in AQP Expression

Most mechanistic evidence derives from rodent models in which constipation is induced with loperamide or diphenoxylate, agents that slow transit and reduce fecal water content. In rats given compound diphenoxylate, proximal-colon AQP3 fell, AQP4 remained unchanged, and AQP8 was undetectable in both groups [37]. In rats with experimentally induced STC, sacral electroacupuncture lowered the elevated distal-colon AQP3 mRNA, reduced intestinal water reabsorption, and raised substance P levels [38]. The herbal formula Liqi Tongbian outperformed the prokinetic mosapride in relieving constipation in a rat model of STC and produced greater reductions in colonic AQP1, AQP3, and AQP4 expression [39]. In loperamide-treated STC mice, the mixed aqueous extract of Aurantii Fructus Immaturus and Magnoliae Officinalis Cortex promoted defecation and motility, lowered colonic AQP3 and AQP4, and raised AQP9 [22].

This variability has several sources. The colonic segment assayed, the species and strain, the duration of opioid exposure, and the detection method all differ between studies. None of these variables is standardized across the field. The aggregate message is nonetheless robust. No single, stereotyped AQP signature of experimental constipation exists, and single-timepoint abundance measurements cannot serve as a reliable surrogate for the constipated state.

### 5.2. Abundance Is Not Flux: The Directionality Problem

Poly-γ-glutamic acid and L-arabinose downregulate AQP3 and AQP4 in mouse models of loperamide-induced STC and diphenoxylate-induced FC, respectively [40,41]. By contrast, Mashiningan extract disturbs colonic water absorption by lowering AQP3 [42]. Other interventions move the same channels in the opposite direction. The hawthorn–probiotic postbiotic raises AQP3 together with ENaC and mucin-2 (MUC2) [43]. Lychee-pulp dietary fiber-bound phenolic complexes upregulate the SCFA–G-protein-coupled receptor (GPR)–enteric nervous system (ENS) pathway and AQPs [44]. Maren pills raise AQP3 and the cystic fibrosis transmembrane conductance regulator (CFTR) while suppressing nuclear factor kappa B (NF-κB) signaling [45]. The two available studies of the same formula, Zengye decoction, illustrate how model and assay differences can reverse an apparent mechanism. One report, combining network pharmacology with in vivo validation, found that the decoction relieved constipation while increasing colonic vasoactive intestinal peptide (VIP), cyclic adenosine monophosphate (cAMP), protein kinase A (PKA), and AQP3, and attributed the effect to a VIP–cAMP–PKA–AQP3 axis [46]. Others found the opposite effect. In the colon of STC rats, AQP3 decreased and AQP9 increased through a miR-10a-5p/*dopamine D2 receptor (Drd2*)/adenylyl cyclase (AC)/cAMP axis [20]. Neither study measured transepithelial water flux directly, so both readouts describe abundance rather than function. The divergence is equally evident among established laxatives. Total diterpenoids of *Euphorbia pekinensis* [47] and emodin [48] both increase colonic AQP3. Kaempferol treatment significantly suppressed colonic AQP4 and AQP8 upregulation, interstitial cells of Cajal (ICC) apoptosis, and oxidative stress in mice with STC [49]. In many of these studies, the AQP change may accompany the therapeutic effect without mediating it, or may represent a homeostatic response to restored luminal hydration. Functional readouts of transepithelial water movement, which are rarely measured in the constipation literature, are therefore required before directional conclusions about AQP-mediated flux can be drawn.

Mice lacking AQP4 exhibit reduced osmotic water permeability in the full-length and proximal colon. However, cecal stool water content remains unchanged, theophylline-induced secretion is unimpaired, and only a marginal difference in defecated-stool water content is observed [50]. This dissociation, in which altered channel-mediated permeability occurs without a commensurate change in net hydration, provides the experimental evidence that channel abundance and channel-mediated flux are not interchangeable measures. This finding also cautions against assuming that an observed change in AQP expression in the constipated colon necessarily alters net water movement, because the same change may be offset by paracellular transport, the lipid bilayer, or compensatory ion transport. Pharmacological tools are also becoming available. DFP00173, a selective AQP3 inhibitor with submicromolar potency, has been applied to the human intestinal cell line HCT8 [51,52], although its effect on net colonic water transport in vivo remains untested. No human genetic variant has yet been robustly associated with FC through an AQP. The strongest causal evidence therefore has yet to emerge, and statements about AQP causality in patients should remain correspondingly restrained. The reported AQP changes and proposed mechanisms of these interventions are compiled in Table 2.

### 5.3. The Mucus Barrier

The mucus layer interposed between the lumen and the epithelium is the first barrier that luminal solutes and microbial products must cross before reaching the AQP-bearing enterocyte. This layer is increasingly recognized as a variable in its own right. The colonic mucus system consists of an outer, loosely adherent layer colonized by commensal bacteria and an inner layer firmly attached to the epithelium. Its thickness and composition are regulated by the goblet-cell program and by microbial signals [56]. In a diphenoxylate-induced mouse model of constipation, the laxative formulation Runchang Tongbian mixture increased MUC2 content while decreasing proximal-colon AQP3 expression [57]. This pattern indicates a coupling between the mucus response and reduced absorptive channel abundance. Because mucus secretion is Ca^2+^-dependent and goblet cells themselves express AQPs, including AQP9 in a mucus-secreting subset [31], the mucus system may modulate AQP function in two ways. It may shape the diffusion environment immediately above the apical membrane, and it may couple channel activity to the hydration demands of mucus secretion. Neither route has been studied quantitatively in the constipated human colon, and the mucus layer remains an under-examined bridge between microbial metabolites and the epithelial water-handling machinery.

## 6. Regulation of Colonic AQPs

Prolonged stool retention changes the osmotic and microbial environment of the lumen, which could, in turn, drive AQP regulation. Conversely, an AQP change that reduces water absorption would reinforce stool dehydration. Cross-sectional abundance data, by design, cannot distinguish between these possibilities. Establishing causality will require longitudinal designs, intervention studies with paired expression and flux readouts, and genetic approaches, such as human cohorts with AQP genotypes or experimental models with inducible, tissue-specific channel deletion.

### 6.1. Inflammation and Cytokine Signaling

Low-grade mucosal inflammation is a recurrent theme in the pathophysiology of FC and STC. In patients with STC, both mast-cell and macrophage populations are expanded in the colonic wall. Mast-cell numbers are increased across all colonic segments, with a relatively uniform distribution [58]. Colonic macrophages are increased in approximately 80% of STC patients, in parallel with reduced miR-128 expression [59]. The inflammatory infiltrate is mechanistically linked to the AQP network. Tumor necrosis factor α (TNF-α) suppresses AQP3 expression in intestinal epithelial cells by inhibiting constitutive AQP3 transcription [60]. In addition, β-carotene has been shown to alleviate inflammation-induced dysregulation of AQP expression via the MAPK signaling pathway [61]. Traditional formulas used in constipation models also combine anti-inflammatory and prokinetic actions. Shouhui Tongbian capsules improved diphenoxylate-induced STC in rats [62]. Jichuan decoction improved loperamide-induced STC by inhibiting the cAMP/PKA/AQPs signaling pathway while restoring inflammatory and gut-microbiota homeostasis [63].

### 6.2. Microbial Metabolites

The gut microbiota now occupies a central position in mechanistic studies of constipation. Arabinoxylan-oligosaccharide has been shown to alter the gut microbiota significantly, increasing Bifidobacterium and decreasing microbial alpha-diversity, in healthy adults with slow gut transit [64]. The evidence linking microbial metabolites to colonic AQP regulation, although derived almost exclusively from rodent models, has grown substantially. SCFAs, the most abundant fermentation products, act both as metabolic fuels and as signaling molecules through GPRs. Their absorption is coupled to Na^+^ and water movement [32,33,65,66]. In a meta-analysis of randomized trials, cereal fibers raised fecal SCFA concentrations in healthy subjects and patients [67]. A human cohort study found that fecal microbial composition differs between constipation subtypes and tracks colonic physiology, lifestyle factors, and psychological status [68]. These observations support a permissive-milieu model. In this model, microbial metabolites determine the luminal osmolarity, the SCFA supply, and epithelial-barrier tonicity within which AQP abundance fluctuates. No single metabolite dictates a single AQP change.

Tryptophan metabolism offers a second, mechanistically richer connection. Tryptophan is metabolized along three principal routes: the kynurenine pathway, serotonin biosynthesis, and microbial indole production. Its metabolites influence gut motility, mucosal immunity, and the gut–brain axis [69]. In postmenopausal women with FC, tryptophan intake was lower and urinary 5-hydroxyindoleacetic acid (5-HIAA) was reduced, whereas the kynurenine pathway and microbial indole derivatives were upregulated [70]. The microbiota thus engages the AQP network at multiple points, through SCFAs, serotonin, and potentially indole derivatives acting on the epithelium.

### 6.3. Neurotransmitters and the Enteric Nervous System

Enteric neurotransmission is the third regulatory axis that converges on colonic AQPs. Serotonin (5-hydroxytryptamine, 5-HT) is the best-characterized link. 5-HT is synthesized by enterochromaffin cells and myenteric neurons and stimulates colonic motility [71]. The opioid–serotonin–AQP3 axis illustrates how a neurotransmitter can act as the intermediary between a drug and a water channel. Morphine increases mucosal 5-HT secretion. The secreted 5-HT is taken up into colonocytes through the serotonin transporter (SERT) and activates peroxisome proliferator-activated receptor γ (PPARγ), which in turn upregulates AQP3 [9].

A VIP–cAMP–PKA–AQP3 sequence has been described in some rodent constipation models. Administration of VIP to constipated rats increased fecal water content together with colonic cAMP, PKA expression, AQP3 mRNA, and AQP3 protein [72]. Network-pharmacology-guided work on the Xiaogan Jianwei formula likewise reported coordinated increases in cAMP, PKA, and AQP3 [53]. These data describe a correlated molecular sequence rather than a demonstrated causal axis. VIP signals through cAMP to stimulate CFTR-mediated secretion, which on its own would move water into the lumen. The increased fecal water content after VIP administration may therefore arise principally from secretion rather than from altered absorptive flux through AQP3. The coincident rise in AQP3 may track the treatment response rather than mediate it. A parallel sequence has been implicated in the action of sciadonic acid, a fatty acid from *Torreya grandis* oil. The sequence involves the 5-hydroxytryptamine (serotonin) receptor 4 (5-HT4), cAMP, PKA, and AQP4. In mice, sciadonic acid reversed the STC-associated decline in the expression of these components [73].

Microbiota-directed and neuromodulatory interventions nonetheless show how AQP changes are embedded in broader neurochemical responses. Colonic AQP4 and AQP8, elevated in diphenoxylate-induced STC, were reduced by *Latilactobacillus sakei* Furu2019 combined with stachyose [74]. These changes occurred alongside parallel changes in c-Kit/SCF signaling. *Lactiplantibacillus plantarum* GUANKE similarly lowered AQP4 and AQP8 and restored 5-HT, substance P, VIP, and nitric oxide levels. It also increased SCF/c-Kit signaling and improved transit [75]. In a mouse model of cathartic colon caused by chronic stimulant-laxative use, *Bifidobacterium bifidum* CCFM1163 restored *tryptophan hydroxylase 1 (Tph1*) expression, colonic 5-HT content, and AQP8 levels [76]. It also repaired tight-junction proteins and enteric nerves and relieved constipation. Across these studies, the AQP changes co-vary with the restoration of neurotransmitter balance and ICC-associated signaling rather than occurring in isolation.

**Figure 2:**
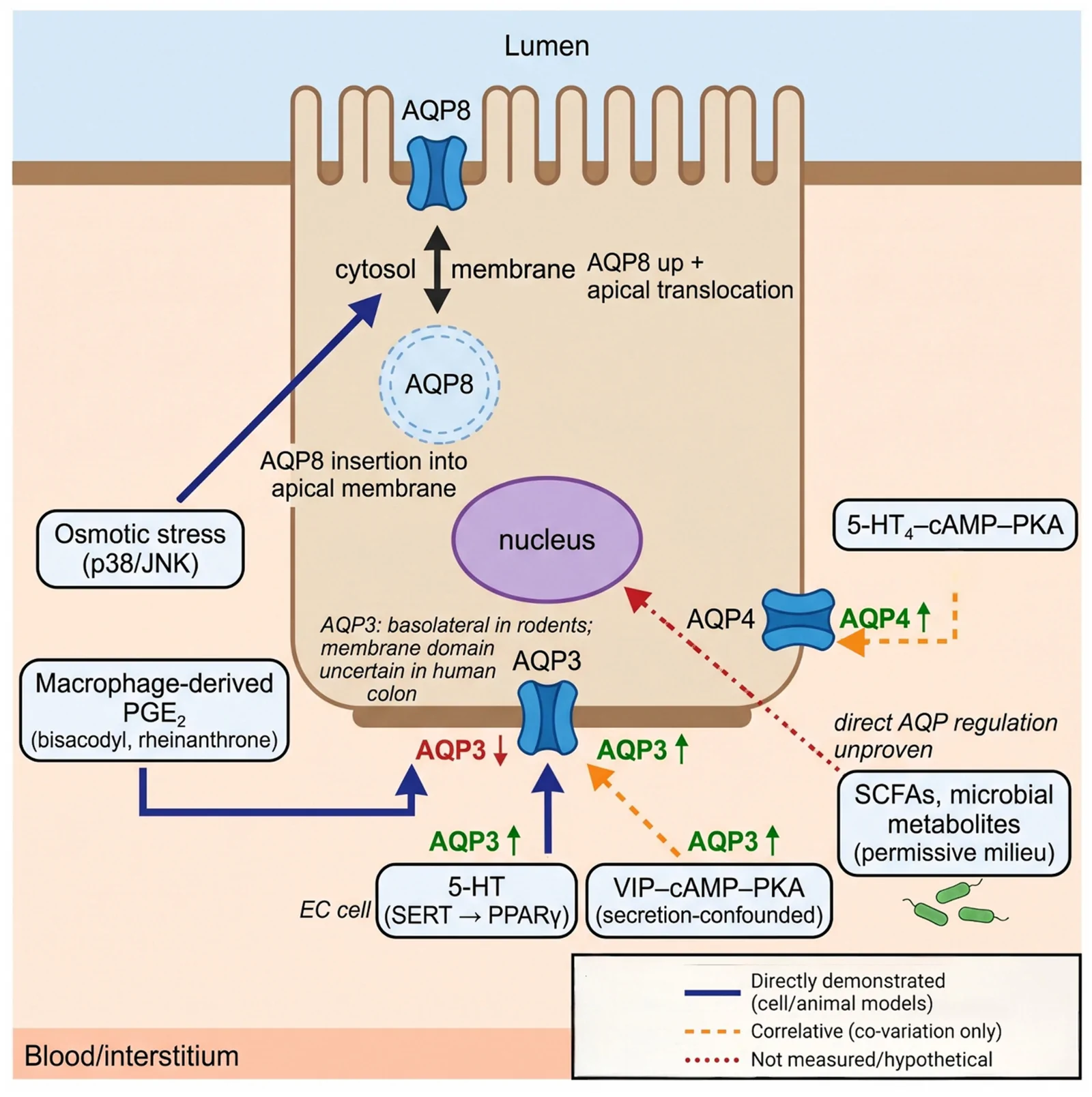
Integrated regulatory network converging on colonic epithelial aquaporins in FC. Macrophage-derived PGE2, the 5-HT–SERT–PPARγ–AQP3 sequence, and osmotic-stress-driven AQP8 translocation are supported by direct experimental manipulation in cell or animal models. The VIP–cAMP–PKA and 5-HT4–cAMP–PKA associations co-vary with secretion and motility rather than demonstrating a causal effect on water flux. Direct transcriptional regulation by microbial metabolites remains speculative. Crucially, even where AQP abundance is robustly altered, the net transepithelial water flux across the constipated colon has not been measured directly, so the physiological consequence of these rearrangements remains inferred rather than demonstrated.

Taken together, the three regulatory axes (low-grade inflammation and cytokine signaling, microbial metabolites, and enteric neurotransmission) converge on a small set of colonic aquaporins, most prominently AQP3, AQP4, and AQP8. They differ sharply, however, in the strength of the evidence linking each signal to a given channel (Figure 2).

## 7. Therapeutic Perspectives

No AQP-targeted therapy has entered clinical testing for FC. Some selective AQP inhibitors have been characterized only in cell-based assays and have not been evaluated for their effects on colonic water transport in vivo. No AQP-directed intervention has been tested in patients. The therapeutic implications are therefore research priorities rather than clinical recommendations, and translational claims should be proportionate to the evidence base.

### 7.1. Laxatives and the AQP Axis

The observation that established laxatives engage colonic AQP regulation links drugs already in everyday use to the water-handling machinery. Bisacodyl, a widely used stimulant laxative, decreases colonic AQP3 expression by directly activating colonic macrophages, which then increase PGE2 secretion. In cultured HT-29 colonocytes, PGE2 exerts a paracrine effect and decreases AQP3 expression to approximately 40% of baseline levels within 30 min. In rats, the same pathway impedes water transport from the lumen to the vasculature [7]. Sennoside A, an active constituent of rhubarb and senna, engages the same pathway. Its metabolite rheinanthrone activates macrophages, raises PGE2, and lowers AQP3. Cyclooxygenase inhibition abolishes both the AQP3 change and the laxative effect [8]. The laxative response to sennoside A is time-dependent and has been tied both to the gut microbiota and to colonic aquaporins [77].

The opposite direction is equally informative. Morphine, the archetypal driver of opioid-induced constipation, raises colonic AQP3 through increased mucosal 5-HT secretion, uptake via SERT, and PPARγ signaling in rodents [9]. The same interpretive problem recurs among the anthraquinone laxatives, a point worth stating explicitly. Emodin raises AQP3 via PKA/p-CREB signaling while increasing fecal water content [48], whereas sennoside A lowers AQP3 via the macrophage–PGE2 pathway [8]. Yet both reportedly relieve constipation. A laxative effect is therefore compatible with changes in AQP levels in either direction, depending on the compound, model, and concurrent ion-transport response. Mechanistic attribution of laxative effects to AQPs is therefore strongest in cell-based systems, where the channel and the driving gradient can be manipulated directly, and remains an inference in whole-animal studies and, a fortiori, in patients.

**Figure 3:**
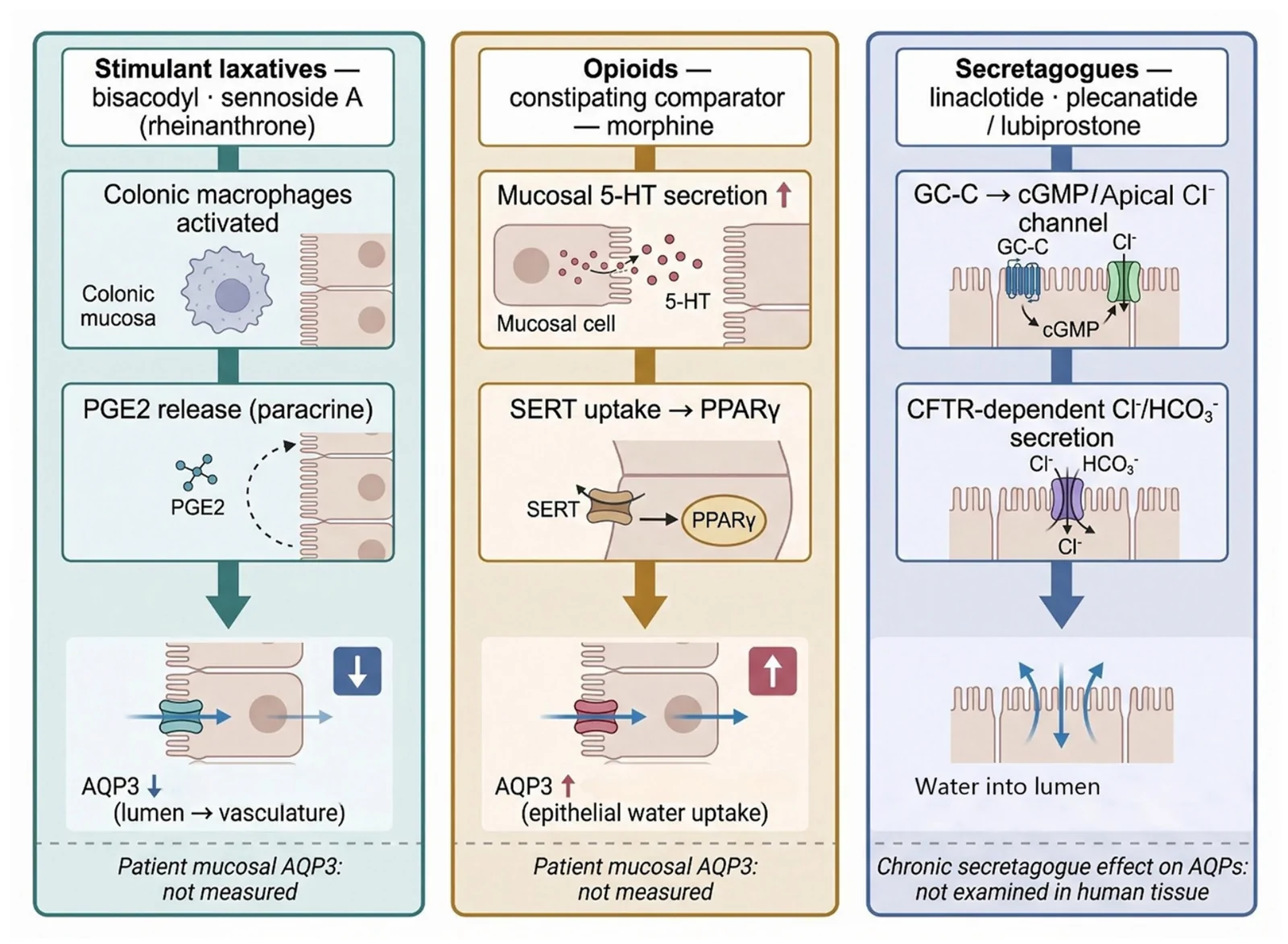
Pharmacologic engagement of colonic AQP3 in opposite directions and the secretagogue complement. Stimulant laxatives (bisacodyl and sennoside A) activate colonic macrophages, whose paracrine PGE2 release downregulates AQP3 in neighboring colonocytes. Opioids (morphine), by contrast, upregulate AQP3 through increased mucosal 5-HT secretion and SERT–PPARγ signaling. Secretagogues drive anion secretion into the lumen, including CFTR-dependent Cl^−^ and HCO_3_^−^ secretion for linaclotide and plecanatide (via guanylate cyclase C) and Cl^−^ secretion through apical Cl^−^ channels for lubiprostone. Abbreviations are listed at the end of the manuscript.

The secretagogue class now central to FC pharmacotherapy illustrates the same solute-first logic from the secretory side. Linaclotide and plecanatide activate guanylate cyclase C on the apical membrane, raising intracellular cyclic guanosine monophosphate and driving CFTR-dependent Cl^−^ and HCO_3_^−^ secretion. Lubiprostone, by contrast, opens apical Cl^−^ channels. In each case, water follows the secreted ions osmotically, traversing the epithelium through both the paracellular route and the transcellular route (lipid bilayer and AQP-mediated). The relative contribution of each route has not been determined in these secretagogue models. Nevertheless, the clinical efficacy of these agents confirms that stimulating anion secretion is sufficient to rehydrate the stool even in patients with slow transit [1,11]. The opposing pharmacologic axes that act on colonic AQP3, together with their relationship to secretagogue-driven secretion, are summarized in Figure 3.

### 7.2. Microbiota-Directed Interventions

Microbiota-directed therapies are among the most intensively studied experimental interventions in constipation research. Their effects on colonic AQP expression have now been reported across numerous rodent models. In loperamide-treated rats, *Bacillus coagulans* restored the loperamide-suppressed aquaporin transcripts and Tph1/5-HT, normalized the loperamide-elevated AQP3 and OCLN proteins, upregulated tight-junction genes (OCLN, ZO-1, CLDN1), and increased cecal acetate and total SCFAs [21]. A konjac-glucomannan and Bifidobacterium animalis F1-7 mixture modulated colonic AQP3 expression in the opposite direction. It downregulated AQP3 while upregulating the 5-HT4 receptor, MUC2, and SCFAs, including butyrate [78]. The one patient-relevant data point, a commercial triple-viable probiotic preparation, accelerated colonic transit and improved symptoms in Rome-IV-defined STC [79]. Its AQP findings, however, originate solely from the accompanying rat model, in which AQP3 and AQP8 mRNA levels increased, whereas Toll-like receptor 2 (TLR2), Toll-like receptor 4 (TLR4), and interleukin-1β (IL-1β) levels decreased. In loperamide-treated mice, both live and heat-killed *Lacticaseibacillus paracasei* K56 relieved constipation, with the live preparation producing greater improvement. Colonic injury was alleviated in both groups, accompanied by reduced AQP2 and AQP3 expression and normalization of 5-HT, substance P, and VIP levels. Both treatments increased Lactobacillus and Lachnospiraceae, promoted butyrate production, and altered tryptophan metabolism [80].

The probiotic and synbiotic studies are predominantly based on rodent models of chemically induced constipation, use diverse strains and dosing regimens, and rarely measure water flux. The AQP changes they report are typically measurements at a single time point whose relation to net transport is unknown. Comparative trials are almost nonexistent, so the relative efficacy of different strains, doses, and formulations cannot be assessed from the current evidence. These findings also illustrate why serotonin and AQP readouts should not be expected to move in lockstep. Neurotransmitter signaling and channel abundance are measured in different compartments, and the microbiota can influence both serotonergic signaling and AQP expression through partly independent routes [9,71,81]. A probiotic may, for example, elevate luminal 5-HT while lowering epithelial AQP3.

### 7.3. Natural Products and Dietary Bioactives

A large and methodologically uneven literature partially attributes the efficacy of traditional formulas and dietary bioactives to modulation of colonic AQPs. The recent rodent studies summarized here illustrate a common template. A plant-derived preparation reverses chemically induced constipation, normalizes stress-associated signaling pathways (e.g., TLR4/MyD88/NF-κB [82], MAPK [54], PI3K/Akt [55]), and shifts AQP3 and/or AQP4 expression toward levels observed in control tissue. Such normalization may involve reversing a constipation-associated upregulation [54,83] or restoring an otherwise suppressed transport system [84]. Siraitiae fructus treatment additionally links the effect to concurrent pulmonary function [82], and Moringa oleifera leaf links it to the ENS [83]. In each case, however, the AQP readout is a single-endpoint abundance measurement. The magnitude of the AQP effect, its segmental distribution, and its contribution relative to the changes in motility and secretion produced by the same interventions are rarely quantified. Many studies use network pharmacology to predict targets and then confirm a subset of the predicted changes, a workflow that is hypothesis-generating rather than validating. Furthermore, whole-plant extracts contain multiple constituents whose individual contributions to the AQP response remain unresolved. Dose–response relationships and time courses are infrequently reported, and few studies control for the effect of restored luminal hydration on AQP expression. This is a potential confounder, because the alterations in AQP expression may be a consequence, rather than a cause, of the improvement in stool water content. The most informative studies measure at least one functional correlate, such as ion transport, barrier function, or motility, in parallel with AQP abundance.

## 8. Conclusions

Human studies document directionally discordant associations between AQP dysregulation and the constipated phenotype. These associations are isoform- and segment-specific. Some channels are upregulated, whereas others are downregulated, depending on the colonic segment and the clinical subtype, and AQP8 additionally undergoes osmotic-stress-driven redistribution. Every human observation to date, however, concerns channel abundance or localization rather than measured water flux, and the cross-sectional designs cannot separate cause from consequence. Experimental models add mechanistic depth but confront the field with a problem of equal interpretive importance. The direction of AQP change is not conserved across models or interventions, and comparable therapeutic benefit can accompany opposite AQP responses. AQPs may set the capacity of the transcellular route without generating the driving force, and a substantial fraction of colonic water traverses the epithelium by the paracellular route.

Regulatory mechanisms of recognized importance in constipation converge on the colonic AQP network, with AQP3 recurring as a common node. These mechanisms include macrophage-derived PGE2 and cytokine signaling, microbial metabolites (including SCFAs and 5-HT), and enteric neurotransmitter cascades such as the VIP–cAMP–PKA and 5-HT4–cAMP–PKA pathways. That drugs already in clinical use engage this node in opposing directions in animal and cell models raises the possibility that colonic AQPs are pharmacologically tractable. Studies that combine AQP abundance with functional readouts of transepithelial water movement, stratify by colonic segment and clinical subtype, and address causal direction are needed before the AQP network can be treated as a therapeutic target in its own right. Until such measurements exist, claims about AQP-mediated water flux in FC should remain provisional, and reports of AQP modulation by experimental interventions would be more readily interpretable if accompanied by ion-transport and mucosal readouts. Within the framework outlined here, AQPs represent a plausible component of the impaired water handling that underlies FC, but whether they are a cause, a consequence, or a modifiable effector will be determined only by studies designed to measure what the channels actually do.

## Figures and Tables

**Table 1:** Summary of aquaporin (AQP) isoforms expressed in the colon.

Isoform	Refs.	Reported Cellular Distribution in the Colon	Reported Change in FC/STC Mucosa
AQP1	[19]	Endothelial cells; enteric glia (subset)	Downregulated (FC)
AQP3	[17,19,23,24,25,26]	Surface enterocytes; apical- or basolateral-predominant depending on species	Upregulated (ascending colon, FC)
AQP4	[18,29]	Colonic mucosa; enteric neurons	Upregulated; correlates with Bristol stool type and colonic transit time (STC)
AQP7	[16,19]	Epithelial cells (low level)	Downregulated (FC)
AQP8	[19,27]	Subapical cytoplasmic vesicles of absorptive cells; osmotic-stress-induced translocation to the apical membrane	Upregulated (FC); translocation reported
AQP9	[17,19,31]	Goblet-cell subset	Downregulated (descending colon, FC)
AQP10	[19,26]	Apical (glycocalyx) compartment of intestinal epithelium (bioinformatically inferred; limited evidence)	Downregulated (FC)
AQP11	[16,19]	Intracellular membranes	Downregulated (FC)

Note: The segment and the direction of change refer to findings reported in the cited studies on functional constipation (FC) or slow-transit constipation (STC). The references also cover localization studies that did not report constipation-related changes. The “direction of change” refers to mRNA and/or protein abundance.

**Table 2:** Reported AQP changes in models of constipation and their proposed mechanisms.

Intervention/Condition	Model	Reported AQP Change	Proposed Mechanism	Refs.
Pharmacological agents				
Bisacodyl (laxative)	Rat (laxative model); HT-29 cells	AQP3 down	Macrophage-derived PGE2; paracrine effect on colonocytes	[7]
Sennoside A (rheinanthrone)	Rat (laxative model); HT-29 cells	AQP3 down	Macrophage-derived PGE2; COX-dependent	[8]
Morphine (constipating comparator)	Rat (morphine-induced constipation); HT-29 cells	AQP3 up	5-HT/SERT/PPARγ	[9]
Dietary bioactives				
Poly-γ-glutamic acid	Mouse STC (loperamide)	AQP3/AQP4 down	ENaC-β down; tight-junction and mucosal repair	[40]
L-arabinose	Mouse FC (diphenoxylate)	AQP3/AQP4 down	5-HT/MTL up, VIP down; tight junctions	[41]
Traditional Chinese medicine formulas				
Zengye decoction (report 1)	Mouse constipation (diphenoxylate compound)	AQP3 up	VIP–cAMP–PKA	[46]
Zengye decoction (report 2)	Rat STC (loperamide)	AQP3 down; AQP9 up	miR-10a-5p/DRD2/AC/cAMP	[20]
Maren pills	Rat STC (diphenoxylate); HT-29 cells	AQP3 up	NF-κB down; AQP3/CFTR up	[45]
Xiaogan Jianwei formula	Rat (loperamide-induced constipation)	AQP3 up	cAMP/PKA/AQP3 axis; MUC2; gut microbiota	[53]
Phytochemicals and teas				
Kaempferol	Mouse STC (loperamide)	AQP4 and AQP8 down	Suppresses oxidative stress and ICC apoptosis (GSTP1-associated)	[49]
Gastrodin	Mouse STC (loperamide)	constipation-associated AQP3/AQP4 upregulation suppressed	MAPK pathway suppression	[54]
Liupao tea	Rat (multi-factor constipation)	AQP3 down	PI3K/AKT	[55]
Probiotics, synbiotics, and postbiotics				
*Bacillus coagulans*	Rat (loperamide-induced constipation)	*Tph1* and AQP transcripts restored	TPH1/5-HT restoration; cecal SCFA; tight junctions	[21]
Hawthorn–probiotic postbiotic	Mouse (loperamide-induced constipation)	AQP3 up	AQP3/ENaC-γ; mucin-2; barrier and microbiota	[43]

Note: All entries derive from rodent models and/or cultured cell lines. None represents a direct measurement in patients, and the directions shown are as reported in the cited studies. The table is representative rather than exhaustive. AC, adenylyl cyclase; GSTP1, glutathione S-transferase P1; ICC, interstitial cells of Cajal.

## Data Availability

No new data were created or analyzed in this study. Data sharing is not applicable to this article.

## Abbreviations

The following abbreviations are used in this manuscript: 5-HIAA, 5-hydroxyindoleacetic acid; 5-HT, 5-hydroxytryptamine (serotonin); 5-HT4, 5-hydroxytryptamine (serotonin) receptor 4; AC, adenylyl cyclase; Akt, protein kinase B; AQP, aquaporin; cAMP, cyclic adenosine monophosphate; c-Kit, stem cell factor receptor (KIT); CFTR, cystic fibrosis transmembrane conductance regulator; COX, cyclooxygenase; CREB, cAMP response element-binding protein; DRA, downregulated in adenoma (Cl^−^/HCO_3_^−^ exchanger, SLC26A3); Drd2, dopamine D2 receptor; ENaC, epithelial sodium channel; ENS, enteric nervous system; FC, functional constipation; GPR, G-protein-coupled receptor; GSTP1, glutathione S-transferase P1; ICC, interstitial cells of Cajal; IL, interleukin; JNK, c-Jun N-terminal kinase; lncRNA, long non-coding RNA; MAPK, mitogen-activated protein kinase; miR, microRNA; MTL, motilin; MUC2, mucin 2; MyD88, myeloid differentiation primary response 88; NF-κB, nuclear factor kappa B; NHE, Na^+^/H^+^ exchanger; NO, nitric oxide; PGE2, prostaglandin E2; PI3K, phosphoinositide 3-kinase; PKA, protein kinase A; PPARγ, peroxisome proliferator-activated receptor γ; SCF, stem cell factor; SCFA, short-chain fatty acid; SERT, serotonin transporter; STC, slow-transit constipation; TLR, Toll-like receptor; TNF, tumor necrosis factor; Tph1, tryptophan hydroxylase 1; VIP, vasoactive intestinal peptide; XCL2, chemokine (X-C motif) ligand 2.
